# Increased Risk of RSV Infection in Children with Down's Syndrome: Clinical Implementation of Prophylaxis in the European Union

**DOI:** 10.1155/2013/801581

**Published:** 2013-06-25

**Authors:** Dianne van Beek, Bosco Paes, Louis Bont

**Affiliations:** ^1^Department of Immunology, University Medical Centre Utrecht, 3508 AB Utrecht, The Netherlands; ^2^Department of Pediatrics (Neonatal Division), McMaster University, Hamilton, ON, Canada L8S 4L8; ^3^Division of Infectious Diseases, Department of Pediatrics, University Medical Centre Utrecht, P.O. Box 85090, 3508 AB Utrecht, The Netherlands

## Abstract

Prospective cohort studies show that Down's syndrome (DS) is an independent risk factor for hospitalization for RSV bronchiolitis. It is unknown whether this observation has been translated into specific management for DS children. The primary goal was to assess the knowledge of healthcare providers in the European Union about RSV infection in DS children and to determine whether it influenced the implementation of prophylaxis. DS caregivers were surveyed using a standardized questionnaire, and country-specific guidelines were obtained. Fifty-three caregivers participated. Thirty-nine (86.7%) had knowledge of the increased risk of severe RSV infection in DS children, and 30 (71.4%) graded that it was important to have a statement on the use of RSV prophylaxis in existing guidelines. Twenty-eight participants had a local DS guideline; hard copies of twelve unique guidelines were obtained. Only one (8.3%) contained a statement on RSV prophylaxis for DS, and five considered such a statement for the next version. *Conclusion*. Most pediatricians had knowledge that DS children have an increased risk of severe RSV infection. Despite the lack of a specific RSV prophylaxis trial in DS, they felt that a statement on RSV prophylaxis in DS guidelines was important, but this was rarely present in current guidelines.

## 1. Introduction

Children with Down's syndrome (DS) suffer more often and more severely from respiratory tract infections. Respiratory syncytial virus (RSV) is a common virus that everybody encounters, which can cause severe infection in high-risk infants. In 2007, studies showed that DS itself is an independent risk factor for severe RSV infection and hospitalization (OR 12.6) [1]. This was confirmed in 2009 and in 2012 [2, 3]. In addition, children with DS have a significantly longer length of hospital stay [4, 5]; they require more frequent mechanical ventilation [4, 5] and sustain a higher mortality rate [5]. RSV cannot be cured and can only be prevented. RSV management includes education of parents on how to prevent infection, the implementation of good hand hygiene, and/or the monthly administration of palivizumab (a monoclonal antibody against the RSV-F protein) during the RSV season [6]. This humanized monoclonal antibody neutralizes the virus as it binds to the antigenic site of the F-fusion protein of RSV. The fusion protein neutralized both RSV serotypes A and B. Palivizumab has become the mainstay for infants with other risk factors for severe RSV bronchiolitis, such as congenital heart disease, chronic lung disease, and prematurity. In those infants, palivizumab has proven to reduce hospitalization rates by 39%–78% [7]. In these populations, the adoption of RSV prophylaxis varies from 25%–100% [8].

The routine use of prophylaxis for children with DS who have additional risk factors for RSV hospitalization is unknown. In addition, it is not known whether the recent knowledge regarding the higher risk of severe RSV bronchiolitis in children with DS has influenced the approach to RSV prophylaxis. In order to determine whether the emerging scientific evidence has impacted RSV prophylaxis in DS infants, both direct and indirect influences merit investigation. An indirect influence is defined as any change in knowledge, awareness, or attitude. Direct influence is any change in behavior, which can include changes in clinical practice, both observed and self-reported. The primary goal of this study was to assess the knowledge of healthcare providers in the European Union (EU) about RSV infection in DS children with and without additional risk factors and to determine whether it has influenced the implementation of RSV prophylaxis. The information will hopefully provide a solid foundation for future research on this topic.

## 2. Materials and Methods

### 2.1. Questionnaire for DS Caregivers

Of the 27 countries in the EU, 25 with more than 1 million inhabitants were selected. Included countries were the following: Austria, Belgium, Bulgaria, Cyprus, the Czech Republic, Denmark, Estonia, Finland, France, Germany, Greece, Hungary, Ireland, Italy, Latvia, Lithuania, The Netherlands, Poland, Portugal, Romania, Slovakia, Slovenia, Spain, Sweden, and the United Kingdom. The target number of participants per country was based on the estimated number of children with DS in that particular country. The estimated number of pediatricians needed in the EU was 68. The sample size was derived based on the combined total of the estimate per country using the population, the birth rate, and the incidence of DS (Table 1). 

Local DS patient organizations in the included countries were asked to provide contact information of pediatricians to participate in the questionnaire. Additional participants were recruited using snowballing (every participant was asked to provide the contact details of two pediatricians specialized in the care of infants with DS). If this resulted in insufficient participations, more potential participants were identified, first, by conducting web-based searches for pediatricians who published articles on DS, RSV, or general pediatric topics and for location for DS clinics preferentially and large hospitals and second, by asking contacts in the countries to identify potential participants. Contact was made using standardized call scripts and electronic mails. Participation was voluntary and non-anonymous.

Indirect knowledge was defined as the percentage of DS caregivers that responded affirmatively to the question “Were you aware that RSV bronchiolitis occurs more often in children with Down's syndrome?” The attitude of DS caregivers regarding the implementation of RSV prophylaxis was determined using a Likert scale that ranged from “very important” (A) to “I do not know” (F) by asking the respective individuals how they felt about the inclusion of a statement on RSV prophylaxis in DS in current guidelines issued by country-specific pediatric advisory bodies. The percentage of DS caregivers answering “very important” was considered a positive attitude. Participants were asked whether they have a local DS guideline, whether this guideline contains a statement on RSV management, and, if not, whether such a statement is being considered for the next version of the guideline. When there was a statement in the guideline about RSV prophylaxis, participants were asked whether and how prophylaxis is reimbursed. Participants were asked which children with DS qualify for RSV prophylaxis locally and whether risk factors are included in the decision to administer RSV prophylaxis. In addition, all participants were asked to provide an estimation of the percentage of children with DS in different subgroups receiving RSV prophylaxis. The familiarity with DS was assessed by asking the participants to estimate the number of children with DS they take care of every month, the incidence of DS in their country and the RSV hospitalization rate for the different subgroups of children with DS.

### 2.2. Down's Syndrome Guidelines

Direct knowledge was defined as the percentage of existing guidelines with a statement on RSV prophylaxis. The local guidelines were obtained via pediatricians, DS patient organizations, and websites. In the obtained local DS guidelines, we evaluated whether the guideline contained the word *RSV* and the word *palivizumab* or *Synagis* or *monoclonal antibody*. We assessed whether the guideline stated that RSV infection is found more often in children with DS and whether the guideline stated that children with DS suffer more severely from RSV infections. We also determined whether the guideline contained a statement on which subgroups of children with DS should receive RSV prophylaxis. Statements were assessed for clarity and the availability of supporting information about RSV infections in children with DS. 

### 2.3. Statistical Analysis

A database was set up in Excel and SPSS 20. Characteristics of the participants and the outcomes were summarized, and results were analyzed using frequencies and proportions. The indirect influence of attitude is portrayed as a radar figure which shows the proportion of the different opinions relative to each other. An overview of the results for the direct influence of existing knowledge is provided in a heat map. Results were analyzed using the Chi-square test or ANOVA where appropriate. Significance was set at a *P* value of ≤0.05. Participants with missing data were excluded from analyses involving the respective variable. 

## 3. Results

### 3.1. Questionnaire for DS Caregivers

The participation rate was 77.9% (53/68; Table 1). There were no participants from Latvia, Bulgaria, and Cyprus. Eight participants (15.1%) were linked to a DS patient organization. Most participants were pediatricians (*n* = 41; 78.8%), two participants were not in the medical field (3.8%), and two were nurses. The mean number of children with DS regularly taken care of within the local hospitals was 171 (SD 288, range 0–1200).

**Figure 1:**
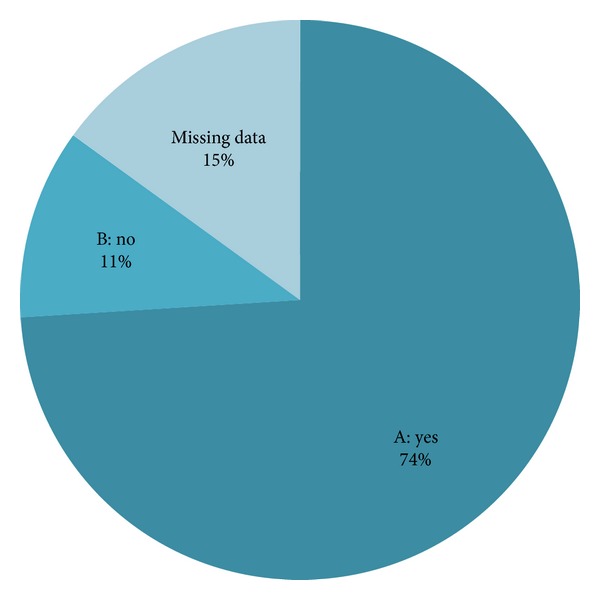
Responses to the following question: Were you aware that RSV bronchiolitis is found more often in children with DS? (A) indicates the answer yes, I was aware. (B) indicates the answer no, but I have heard of the disease. (Missing data) indicates the percentage of participants who did not answer this question.

**Figure 2:**
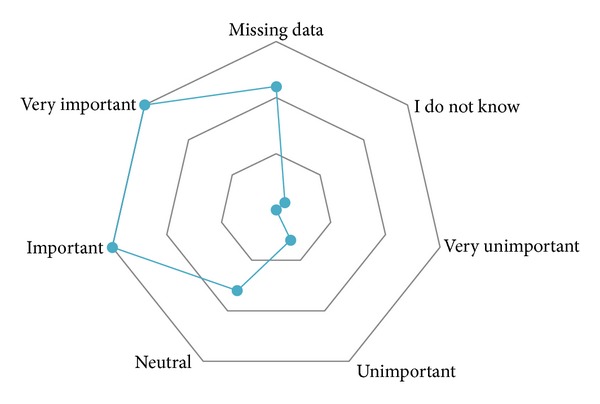
Responses to the following question: How do you feel about a statement on RSV palivizumab use in guidelines/protocols for management of children with DS? The responses are shown in relation to each other.

All participants had knowledge of RSV bronchiolitis, six (13.3%) were not aware of the increased risk in DS patients, and the majority (*n* = 39, 86.7%) were aware that RSV bronchiolitis occurs more often in children with DS (Figure 1). The source of this knowledge was almost always the scientific literature (*n* = 32; 82.1%). Data were not available from eight participants. The majority (*n* = 30, 71.4%) of participants reported that a statement on RSV prophylaxis in DS guidelines is important or very important, whereas only three (7.1%) reported this to be unimportant; seven participants were neutral (16.7%), and one participant did not know whether such a statement was relevant (2.4%) (Figure 2). Twelve participants did not provide responses to this question.

Five participants (11.1%) stated that they have a local DS guideline with a statement on RSV prophylaxis, and according to five participants such a statement is being considered for the next version of the guideline. Three participants responded that they never use palivizumab for children with DS (5.6%), and two used palivizumab for all children with DS (3.8%). Thirty one (58.5%) and 25 (47.2%) of the participants supported the implementation of RSV prophylaxis in DS patients with congenital heart disease or prematurity, respectively. Participants responded most commonly that DS children with CHD and those born prematurely (<32-week gestational age) received RSV prophylaxis *regularly* (defined as >10% receiving prophylaxis) (*n* = 21, 39.6%; *n* = 18, 34.0%; Figure 4). Preventative hygiene and education is promoted by only 11 (20.8%) of the participants. 

### 3.2. DS Guidelines

**Figure 3:**
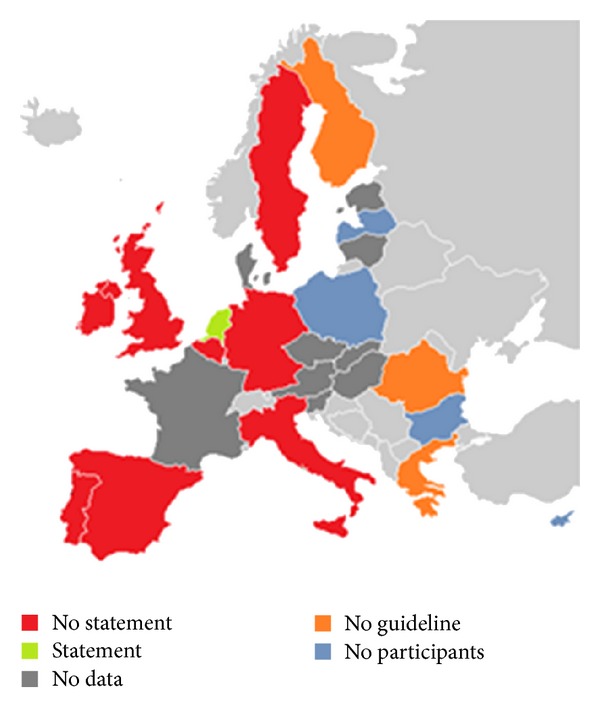
RSV statement in guidelines. Heat map according to the responses in the questionnaire about the presence of a guideline and the presence of a statement in the guideline.

**Figure 4:**
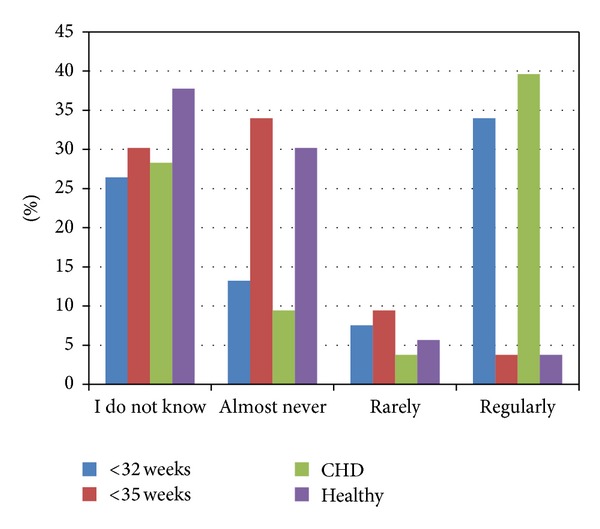
Answers to the following question: Can you give an estimation of the percentage of children in your country/state receiving RSV prophylaxis among the following groups? Answer options are given as follows: I do not know, almost never (0%–5%), rarely (5%–10%), regularly (>10%); <32 weeks: children with DS born prematurely gestational age <32 weeks (missing data: *n* = 10, 18.9%); <35 weeks: children with DS born prematurely gestational age <35 weeks (missing data: *n* = 12, 22.6%), CHD: children with DS and congenital heart disease (missing data: *n* = 10, 18.9%); healthy: otherwise healthy children with DS (missing data: *n* = 12, 22.6%).

Not all guidelines were obtained, mostly because the guideline was not available or not in English. A copy was obtained of twelve unique guidelines. Guidelines were obtained from Belgium, Germany, Ireland, and The Netherlands, and two distinct ones were obtained from the group representing Italy, Spain, the United Kingdom, and Portugal. The guidelines and publication dates varied from 2001 to 2011. The presence of a DS statement was confirmed in only one guideline (The Netherlands'; Figure 3); this was also the only guideline that contained the words RSV and palivizumab or Synagis or monoclonal antibody. 

### 3.3. Factors Associated with Implementation

Participants in the questionnaire were asked to provide their best estimate of the incidence of RSV hospitalization. Hospitalization rates did not significantly influence implementation of a guideline for RSV prophylaxis. An estimated incidence of DS of  >1 : 800 live births was arbitrarily considered a high incidence of DS. A high incidence of DS was associated with having a DS guideline (*n* = 5, 83.8% compared with *n* = 22, 59.5%). 

## 4. Discussion

Children with Down's syndrome have a very high risk of severe RSV bronchiolitis. Implementation of this knowledge in the care for these children, however, is unknown. We found a statement on the use of RSV prophylaxis in only one (8.3%) of the twelve guidelines. According to five DS caregivers who participated in the questionnaire, such a statement on the use of RSV prophylaxis is being considered for the next version of the local guideline. Overall, there was a high awareness of the increased risk of RSV infection for children with DS among DS caregivers, and the majority of participants felt that a statement on RSV prophylaxis in DS medical guidelines was very important. 

This is the first study to investigate the breadth of knowledge regarding RSV infection among DS healthcare providers and the impact of their knowledge on the implementation of RSV-specific prophylaxis in the countries of the European Union. In this survey, only 2 to 21 (3.8%–39.6%) of the participants estimated that RSV prophylaxis is given *regularly* to children with DS for a defined indication. The lower rate of prophylaxis might explain why the hospitalization rate is estimated higher, most commonly >10%, compared with the expected rate of 1%-2% with palivizumab use. The gauged hospitalization rate by the caregivers for healthy DS children closely aligns with the literature with 5%–10% being the most common answer. Paes et al. [9] observed a 4-fold proportional increase, from 2006–2010, in the number of patients receiving palivizumab for off-label underlying medical disorders of which the largest increase was evidenced in the DS group that comprised 20.3% of the entire cohort [9]. Although the study did not distinguish DS infants with or without risk factors, more recently Zachariah et al. confirmed that DS infants independent of risk factors are at higher risk for hospitalization following RSV lower respiratory tract illness [3]. Our study also shows that palivizumab is being used “off-label” in the EU for children with DS without additional risk factors. Two (3.8%) of the participants estimated that >10% of the healthy children with DS receive RSV prophylaxis.

Principles governing the lack of a specific RSV management guideline for children with DS (direct influence) cannot be determined from this research. Anderson et al. determined that lack of understanding of the severity of RSV infection is a major obstacle for successful implementation of RSV prophylaxis [10]. In our study, the participants did show an awareness of the increased risk of severe RSV infection in children with DS. Since the indirect influence appears positive and indirect and direct influences are not significantly associated, additional factors must impact the implementation of prophylaxis. One factor may be patient agreement to receive prophylaxis and ongoing commitment to complete the course of injections throughout the RSV season. Compliance with palivizumab is improved with home-based administration compared with office-based administration [8]. Anderson et al. [10] also identified that pediatricians wanted more educational materials about RSV disease for both personal and family edification and felt that parent reminders might improve compliance and the implementation of a successful program. Warren et al. [11] optimized RSV prophylaxis using a provincial approval system and reached 100% of eligible children with congenital heart disease. Our research does not address potential patient factors impairing the implementation of prophylaxis, and we did not investigate how cost of the monoclonal antibody may impede the adoption of prophylaxis across the EU.

Strengths and limitations of this study merit further discussion. First, conducting a patient-based study to determine reasons that may govern the implementation of prophylaxis was not feasible. Second, a nonvalidated questionnaire was used. Third, the proportion of valid responses in the questionnaire was lower for participants with an RSV statement in their local guideline. Fourth, not all available guidelines were collected, and proportionally more English than non-English guidelines were obtained. However, participants from 22 different countries took part in this research. Fifth, both the direct and the indirect influences have been addressed using the questionnaires and complementary local guidelines. Several questions had a considerable proportion of missing data. Although this is a methodological limitation, it also provides additional information regarding which questions were most difficult to answer. 

RSV bronchiolitis is a major health issue in children with Down's syndrome, as it accounts for 17.6% [5] of all DS admissions to hospital (compared with 7%–9% overall [12]). The incidence of hospitalization for RSV infection in children with DS in large cohorts is 9.9%–17.6% [1, 5], which is higher than the hospitalization rate (1%) in the normal population. Moreover, it has recently been shown that palivizumab treatment reduces the wheezing days in otherwise healthy-late preterm infants [13]. This might be of particular interest for children with DS, as they have an increased risk of recurrent wheezing, although the role of RSV in recurrent wheezing in DS is not yet established [14].

Despite this knowledge, RSV management has not been integrated into the care of children with DS. The results of this study can be considered a baseline for future research on this topic. The results may be instrumental in designing and implementing a prophylaxis program aiming to improve the care of children with DS through the prevention of RSV bronchiolitis. 

Five years after the first publication on the risk of severe RSV bronchiolitis in children with DS, the majority of the participating DS caregivers in the EU are aware of the increased risk of severe RSV bronchiolitis for children with DS. Despite the absence of a randomized placebo-controlled trial, in general the implementation of specific RSV management for this group was deemed to be very important. Nevertheless, virtually no guidelines in the EU have a statement on the management of RSV bronchiolitis in this high-risk population. 

## Figures and Tables

**Table 1:** Number of pediatricians needed in the European Union.

Country	Population^1^	Birth rate 2011^2^	Incidence DS^3^	Participations
Austria	8.2	9	15,66	1
Belgium	10.4	10	16	3
Bulgaria	7.1	9	12,29	0
Cyprus	1.1	0*	17,12**	0
The Czech Republic	10.2	9	18,94	1
Denmark	5.6	10	16,92	2
Estonia	1.3	10	17,12**	1
Finland	5.3	10	24,53	2
France	65.3	12	29,42	7
Germany	81.5	8	26,31	6
Greece	10.8	9	17,12**	1
Hungary	9.9	10	13,32	1
Ireland	4.7	16	26,82	3
Italy	61.0	9	16,28	5
Latvia	2.2	10	17,12**	0
Lithuania	3.5	9	17,12**	1
The Netherlands	16.8	10	15,19	1
Poland	38.4	10	15,06	0
Portugal	10.8	10	7,61	4
Romania	21.9	10	17,12**	1
Slovakia	5.5	10	17,12**	1
Slovenia	2.0	9	17,12**	1
Spain	46.8	11	27,2	2
Sweden	9.1	10	29,3	2
The United Kingdom	62.9	12	28,09	7

Total				53

^1^Population in millions (http://www.europa-nu.nl/).

^2^Births per 1000 populations (http://www.indexmundi.com/).

^3^Incidence per 10.000 births based on average prevalence measured between 1980 and 2009 (http://www.eurocat-network.eu/).

*Birth rate in Cyprus is not published.

**DS incidence is not published; average of all in the database was utilized.

## Abbreviations

DS: Down's syndrome
EU: European Union
RSV: Respiratory syncytial virus.

## References

[1] Bloemers BLP, Van Furth AM, Weijerman ME (2007). Down syndrome: a novel risk factor for respiratory syncytial virus bronchiolitis—a prospective birth-cohort study. *Pediatrics*.

[2] Kristensen K, Stensballe LG, Bjerre J (2009). Risk factors for respiratory syncytial virus hospitalisation in children with heart disease. *Archives of Disease in Childhood*.

[3] Zachariah P, Ruttenber M, Simões EAF (2012). Down syndrome and hospitalizations due to respiratory syncytial virus: a population-based study. *Journal of Pediatrics*.

[4] Fjaerli H-O, Farstad T, Bratlid D (2004). Hospitalisations for respiratory syncytial virus bronchiolitis in Akershus, Norway, 1993–2000: a population-based retrospective study. *BMC Pediatrics*.

[5] Megged O, Schlesinger Y (2010). Down syndrome and respiratory syncytial virus infection. *Pediatric Infectious Disease Journal*.

[6] Paes BA, Mitchell I, Banerji A, Lanctôt KL, Langley JM (2011). A decade of respiratory syncytial virus epidemiology and prophylaxis: translating evidence into everyday clinical practice. *Canadian Respiratory Journal*.

[7] Connor EM (1998). Palivizumab, a humanized respiratory syncytial virus monoclonal antibody, reduces hospitalization from respiratory syncytial virus infection in high-risk infants. *Pediatrics*.

[8] Frogel MP, Stewart DL, Hoopes M, Fernandes AW, Mahadevia PJ (2010). A systematic review of compliance with palivizumab administration for RSV immunoprophylaxis. *Journal of Managed Care Pharmacy*.

[9] Paes B, Mitchell I, Li A, Lanctôt K (2012). Respiratory hospitalizations and respiratory syncytial virus prophylaxis in special populations. *European Journal of Pediatrics*.

[10] Anderson KS, Mullally VM, Fredrick LM, Campbell AL (2009). Compliance with RSV prophylaxis: global physicians' perspectives. *Patient Preference and Adherence*.

[11] Warren A, Langley JM, Thomas W, Scott J (2007). Optimizing the delivery and use of a new monoclonal antibody in children with congenital heart disease: a successful provincial respiratory syncytial virus prophylaxis program. *Canadian Journal of Cardiology*.

[12] Eidelman AI, Megged O, Feldman R, Toker O (2009). The burden of respiratory syncytial virus bronchiolitis on a pediatric inpatient service. *Israel Medical Association Journal*.

[13] Blanken MO, Rovers M, Molenaar JM (2013). Respiratory syncytial virus and recurrent wheeze in healthy preterm infants. *New England Journal of Medicine*.

[14] Bloemers BLP, Van Furth AM, Weijerman ME (2010). High incidence of recurrent wheeze in children with down syndrome with and without previous respiratory syncytial virus lower respiratory tract infection. *Pediatric Infectious Disease Journal*.

